# An approach to evaluate the effect of inflammatory microvesicles on Ca^2+^ handling in human-induced pluripotent stem cell-derived cardiomyocytes

**DOI:** 10.3389/ebm.2025.10461

**Published:** 2025-08-28

**Authors:** Dania Fischer, Mishkaat Sha’sha’a, Judith Schenz, Aycan Tayan, Christina Mertens, Sebastian O. Decker, Nadia Gallenstein, Maximilian Dietrich, Trim Lajqi, Anna Hafner, Markus A. Weigand, Nina D. Ullrich

**Affiliations:** ^1^ Department of Anesthesiology, Medical Faculty Heidelberg, Heidelberg University, Heidelberg, Germany; ^2^ DZHK (German Centre for Cardiovascular Research), Partner Site Heidelberg, Heidelberg, Germany; ^3^ Department of Cardiovascular Physiology, Heidelberg University, Heidelberg, Germany; ^4^ Center for Translational Biomedical Iron Research, Department of Pediatric Oncology, Immunology, and Hematology, University of Heidelberg, Heidelberg, Germany; ^5^ Department of Neonatology, Heidelberg University Children’s Hospital, Heidelberg, Germany; ^6^ Department of Physiology, University of Bern, Bern, Switzerland

**Keywords:** cardiomyopathy, sepsis, cardiomyocytes, calcium transients, microvesicles

## Abstract

Microvesicles (MV) isolated from septic individuals were observed to impact systemic hemodynamics and cardiac function. The aim of this *in vitro* study was to analyze the effects of TNFα-induced endothelial MV (TMV) and MV from septic patients (SMV) on beating frequency and Ca^2+^ transient kinetics of human-induced pluripotent stem cell-derived cardiomyocytes (hiPSC-CM). MV were isolated from supernatants of TNFα-stimulated primary human pulmonary microvascular endothelial cells (HPMEC) and plasma from 20 sepsis patients by ultracentrifugation and quantified using flow cytometry. Spontaneous Ca^2+^ transients were measured in hiPSC-CM using the Ca^2+^-sensitive ratiometric indicator fura-2 at different time points of incubation with different MV concentrations. At 16 h of incubation, higher MV concentrations showed significant differences, especially regarding decay and beating frequency. Despite high variability, at 10 × 10^6^ MV/mL and 16 h of incubation, TMV significantly decreased frequency compared to control MV (CMV). SMV from septic patients did not reveal any significant effects on Ca^2+^ transients under these experimental settings. MV isolated from control or TNFα-treated HPMEC affected Ca^2+^ handling and spontaneous activity of hiPSC-CM, however, the measured effects were not consistent throughout the different conditions. Further refinement of the experiment conditions is needed to specify the exact conditions for crosstalk between endothelium-derived MV and cardiomyocytes.

## Graphical Abstract

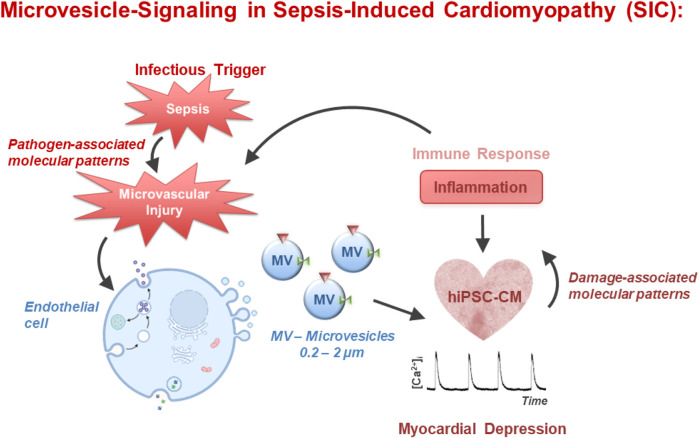

## Impact statement

Given the established role of extracellular microvesicles in cellular communication and their potential impact on various physiological and pathological processes, we believe our findings are highly relevant to the scope of your journal. Our findings suggest that while microvesicles can modulate cardiac function, the precise conditions under which this crosstalk occurs require further refinement. This study offers valuable insights into the complex interactions between endothelium-derived microvesicles and cardiomyocytes, highlighting the need for more detailed investigation into the conditions that facilitate this interaction. This manuscript contributes to the growing body of knowledge in this field and spur further research into the therapeutic and diagnostic potential of microvesicles in sepsis and other cardiovascular conditions.

## Introduction

Sepsis is a life-threatening clinical syndrome that arises from a severe disturbance in the body’s reaction to infection. Parker et al. were the first to describe the association of newly developed myocardial dysfunction and its impact on mortality in patients with sepsis [1]. Since then, cardiac dysfunction has been recognized as a significant comorbidity in sepsis, termed septic cardiomyopathy. The prevalence and impact of septic cardiomyopathy can vary, but it is estimated that a significant proportion of patients with severe sepsis or septic shock develop cardiac dysfunction [2]. Septic cardiomyopathy is a complex condition, and there are still multiple aspects that are not fully understood [3]. Extracellular vesicles (EV) may participate in the pathogenesis of sepsis and septic cardiomyopathy in multiple ways. EV have been detected in the circulation of sepsis patients [4, 5]. EV are membrane vesicles released during cell activation, currently being considered as diagnostic biomarkers, mediators, or even therapeutic agents of sepsis [6, 7]. They are known to be active in cell-to-cell communication and can be differentiated by size and surface markers [8]. There are three types of EV, namely the submicron-size microvesicles (MV), the nanometer-size exosomes, and apoptotic bodies, which measure up to several micrometers in diameter [9].

Azevedo et al. reported that platelet-derived exosomes from septic shock patients induce myocardial dysfunction in isolated heart and papillary muscle preparations [10]. This negative inotropic effect was fully reversible upon withdrawal of exosomes. Mortaza et al. found that rats with sepsis induced by peritonitis exhibited a specific phenotype of MV derived from leukocytes. Inoculation of these MV in healthy rats reproduced hemodynamic, septic inflammatory patterns associated with oxidative and nitrosative stress [11]. Furthermore, Zhang et al. observed that H9c2 cells, derived from embryonic rat cardiomyocytes, exhibited reduced cell viability and increased cell apoptosis and reactive oxygen species production when treated with EV derived from hypoxia/reoxygenation-treated human umbilical vein endothelial cells [12].

Sepsis-induced endothelial dysfunction and capillary leakage lead to impaired myocardial perfusion and tissue hypoxia, which further compromise cardiac function. Moreover, sepsis triggers an overwhelming release of pro-inflammatory cytokines. Tumor necrosis factor-α (TNFα) is one important pro-inflammatory cytokine upregulated in many inflammatory diseases and is a potent inducer of endothelial cell (EC)-derived MV formation [13]. These MV and ECs were shown to be involved in the pathophysiological mechanisms of sepsis and septic shock [14]. Moreover, exosomes from patients with septic shock convey miRNAs and mRNAs related to pathogenic pathways, including inflammatory response, oxidative stress, and cell cycle regulation [15]. It was also shown that miRNAs derived from neutrophil-derived EV play an important role in sepsis-associated cardiomyopathy. These miRNAs were shown to induce hypoxia inducible factor-1 (HIF-1) signaling to elicit development of septic cardiomyopathy in septic patients [16].

Based on the above mentioned clinical and experimental studies, we tested the hypothesis that MV isolated from septic patients affect cardiomyocyte contractility. To test this, we employed human-induced pluripotent stem cell-derived cardiomyocytes (hiPSC-CM) as our model system. Initially, we examined MV generated *in vitro* under stress conditions (induced by TNFα) by microvascular endothelial cells. These MV were applied directly to cardiomyocytes within a well-controlled experimental framework. We analyzed various MV concentrations and defined incubation times to assess their impact on cardiac Ca^2+^ handling. Since electromechanical coupling in cardiomyocytes is governed by intracellular Ca^2+^, and Ca^2+^ transients are tightly regulated, even minor effects on contractility can be detected through changes in Ca^2+^ transient kinetics. Therefore, any alterations in Ca^2+^ homeostasis directly influences cardiomyocyte contractility. The goal of this *in vitro* study was to systematically investigate the effects of TNFα-induced endothelial MV and MV from septic patients on Ca^2+^ handling of hiPSC-CM.

## Materials and methods

### Cell culture

#### Endothelial cells

Primary human pulmonary microvascular endothelial cells (HPMEC) were commercially obtained from Promocell (C-12281, Heidelberg, Germany). Cells were cultivated under standard cell-culture conditions (37°C, 5% CO_2_) in endothelial cell growth medium (MV2, C-22022) complemented with supplement-mix (C-39226). The medium was exchanged every two to 3 days. When cells reached 60%–70% confluency, they were split according to the manufacturer’s instruction using the detach kit (C-41210, Promocell). Accordingly, cells were washed with HEPES (C-40010) and then detached using 0.04% Trypsin/0.03% EDTA (C-41010). After 3 min of centrifugation at 250 *g* and 37°C, the cell-pellet was resuspended. Cells were counted and 500,000 cells were added to a T75 flask prefilled with warm medium. Cells were expanded up to passage 7. At 90% confluency, cells were incubated with 100 ng/mL recombinant human TNFα (300-01A, PeproTech, Hamburg, Germany) for 24 h with a respective control of medium-only cells. Thereafter, supernatants were collected and snap-frozen for later MV isolation of TNFα-stimulated (TMV) and control MV (CMV).

#### Stem cell culture

Human-induced pluripotent stem cells (hiPSC) were provided by Dr. Cyganek, Stem Cell Unit Göttingen, University Medical Center Göttingen. Using the integration-free Sendai virus as described before by Rössler et al., the wild type hiPSC-line UMGi014-C clone 14 was generated from dermal fibroblasts [17]. Cells were seeded in StemFit Basic04 Complete Medium (Basic04CT, Nippon Genetics, Düren, Germany) containing the ROCK-inhibitor thiazovivin (1:1000, 72254, StemCell Technologies, Cologne, Germany) for 24 h. For maintenance of the culture, medium was changed every other day. At 70% confluency, cells were passaged. Standard cell culture plates were coated with 1:300 Matrigel (Corning, Berlin, Germany) in DMEM/F-12 (Gibco, Darmstadt, Germany) for at least 30 min (37°C, 5% CO_2_). Cells were detached using 0.5 M EDTA (15575020, Thermo Fisher scientific, Darmstadt, Germany) for 7–10 min. After centrifugation at 25 *g* for 5 min, the cell pellet was resuspended in StemFit/thiazovivin medium and replated both in a 12-well plate for differentiation (1:20 to 1:40) and in a 6-well plate for maintenance (1:10 to 1:20).

#### Cardiac differentiation

**FIGURE 1:**
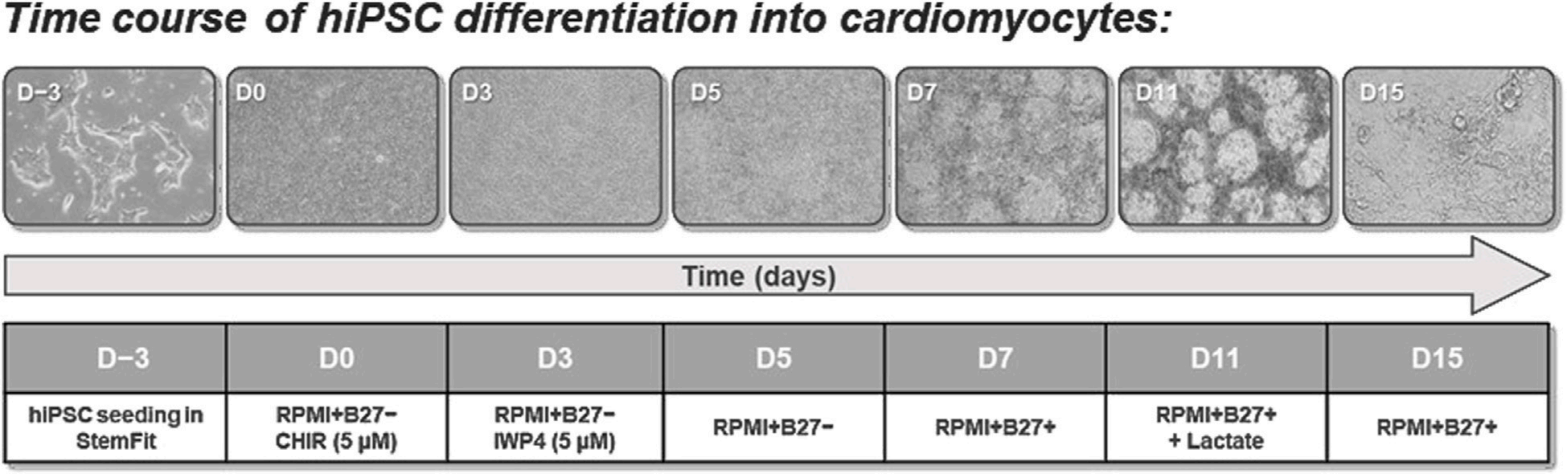
hiPSC differentiation to cardiomyocytes (hiPSC-CM). hiPSC were seeded three days before (D-3) start of differentiation (D0) and grown to colonies of about 70% confluency. Starting with D0, the Wnt signaling pathway was subsequently first activated (CHIR) and then inhibited (IWP4) to induce mesoderm and cardiogenic determination. From D9 onward, cells were beating and further metabolically selected using lactate. After selection, cells were kept in maintenance medium (RPMI+B27+) until further use.

The differentiation protocol is based on a Wnt/β-catenin signaling pathway modulation [18]. The procedure is described in Figure 1. At 95% confluency, the differentiation process was induced (day 0). hiPSC were treated with 5 µM CHIR99021 (72054, Stemcell Technologies) in RPMI 1640 (61870010, Life Technologies, Thermo Fisher Scientific) containing 11 mM glucose and B27 supplement without insulin (1:50, A18956, Gibco, Thermo Fisher Scientific). After 24 h, cells were maintained in diluted CHIR (2.5 µM) to improve the differentiation efficiency [18]. On day 3, medium was changed, and the Wnt pathway inhibitor IWP-4 (5 μM, 72552, StemCell Technology, Cologne, Germany) was added for 48 h, with a medium change on day 5. On day 7, the medium was switched to RPMI-B27+insulin (1:50, 17504, Gibco, Thermo Fisher Scientific) and changed every other day. Once contractions were detected, metabolic selection of cardiomyocytes was initiated by replacement of glucose with sodium lactate (5 mM, L4263, Sigma-Aldrich, Merck) in RPMI 1640 + B27+insulin without glucose (11879020, Gibco). After 5 days of selection, hiPSC-CM were maintained in RPMI+B27+insulin.

#### Cardiomyocyte cell culture

Day 16 post differentiation, hiPSC-CM were seeded on glass bottom dishes (GBD, P35G-1.5-14-C, MatTEK, Life Sciences, Bratislava, Slovakia) for experimental purposes. Cells used for all experiments were differentiated from hiPSC passages 40-54. GBD were coated with Matrigel as described above. Splitting medium contained RPMI+B27+insulin without glucose (Life Technologies), 10% foetal bovine serum (Gibco), 0.1% thiazovivin (72254, StemCell Technology), and 1% penicillin/streptomycin (P4458, Sigma-Aldrich). Cells were rinsed twice with PBS (D8537, Sigma-Aldrich) and incubated with TrypLE Express (1-5x, 12605, Gibco). After 10 min of incubation (37°C, 5% CO_2_), cells were gently detached. Cells were centrifuged at 6.2 *g* for 10 min, resuspended in splitting medium, counted, and seeded at 30,000 cells per dish on a GBD. Within 24 h, the medium was changed using RPMI+B27+insulin and changed every second day thereafter.

### Sepsis and control plasma acquisition

Ethical approval for this study (reference number S-664/2020) was provided by the Ethical Committee of the Medical Faculty of Heidelberg University. The study was registered at the German Clinical Trial Register (DRKS00023301). Adult participants with abdominal, respiratory, or urinary tract sepsis were recruited within the first 24 h of sepsis onset. Inclusion criteria for patients with sepsis were applied according to Sepsis-3 criteria [19]: life-threatening organ dysfunction caused by a suspected or proven infection, an acute increase in total SOFA score of ≥2 points, onset <24 h, and age ≥18 years.

Whole blood was drawn on day 1 and centrifuged for 10 min at 2,000 *g* at room temperature. The plasma was further centrifuged for 15 min at 800 g and the supernatant was collected for isolation of septic MV (SMV). As a control for SMV, plasma was obtained from a plasmapheresis procedure, during which 12 freshly frozen plasmas from healthy donors were used for plasma exchange of a patient with ABO incompatibility before organ transplantation to gain CMV (Plasmapheresis CMV: (P)CMV). Both sepsis and plasmapheresis plasma were stored at −80°C.

### MV preparation and incubation

Human primary microvascular endothelial cells (HPMEC) were cultured up to a maximum 7 passages to preserve the endothelial phenotype *in vitro*. Cells were incubated with TNFα (100 ng/mL). Unstimulated HPMEC served as control. After 24 h of incubation, medium was collected and MV were isolated and characterized as described below.

#### MV isolation

The following steps were conducted at 4°C. Defrosted plasma and cell culture supernatants were centrifuged for 15 min at 500 *g*. The supernatant was collected and centrifuged for 20 min at 4000 *g*. The supernatant of these samples was ultracentrifuged for 90 min at 100,000 *g*. Afterwards, the pellet was resuspended in PBS and centrifuged at 100,000 *g* for another 90 min. Finally, the MV pellet was collected and resuspended in PBS. All samples were quantified and stored at −70°C to preserve MV count.

#### MV characterisation and quantification

Flow cytometry was performed using the BD FACSLyric flow cytometer (BD Bioscience, Franklin Lakes, NJ, USA) with the accompanying BD FACSuite software.

**FIGURE 2:**
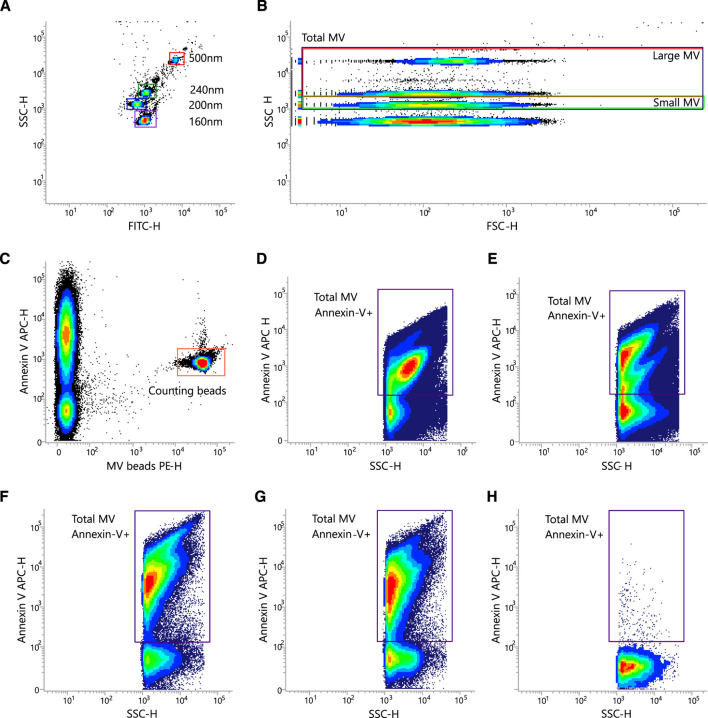
Representative gating strategy for quantification of MV. **(A–E)** Flow cytometer settings and gating strategy for MV quantification with Megamix-Plus SSC and MV-Counting Beads (0.3 µm sized PE fluorescent beads). Shown are density/dot blots from representative measurements. SSC-H vs. FITC-H **(A)** and SSC-H vs. FSC-H **(B)** density blot showing the location of the Megamix-Plus SSC beads. Beads were used to set up the SSCH, FITC-H, and FSC-H PMT voltage **(A,B)**, the MV gates **(B)**, and the SSC threshold for MV analysis. **(C)** Beads with a known concentration were spiked into all MV samples. Shown is a representative analysis of an MV isolate as SSC-H vs. FSC-H dot blots illustrating the Annexin V-stained MV within the prior defined (see **(B)**) MV gates. **(D-G)** Four examples of the gating strategy to characterize the MV population. **(H)** Negative control after addition of 20% Triton X-100.

All materials were filtered through a 0.22 μm sterile filter. Megamix-Plus SSC beads (7803, Biocytex, Marseille, France) were used to adjust the flow cytometer settings for standardized MV analysis according to the manufacturer’s instructions (Figure 2). MV were stained with Annexin-V APC (550474, BD Bioscience). MV were defined as Annexin-V positive events. MV-Counting Beads with a known concentration were spiked into each MV sample for quantification. The negative control was performed by adding Triton X-100.

#### Pierce protein assay for protein concentration

**FIGURE 3:**
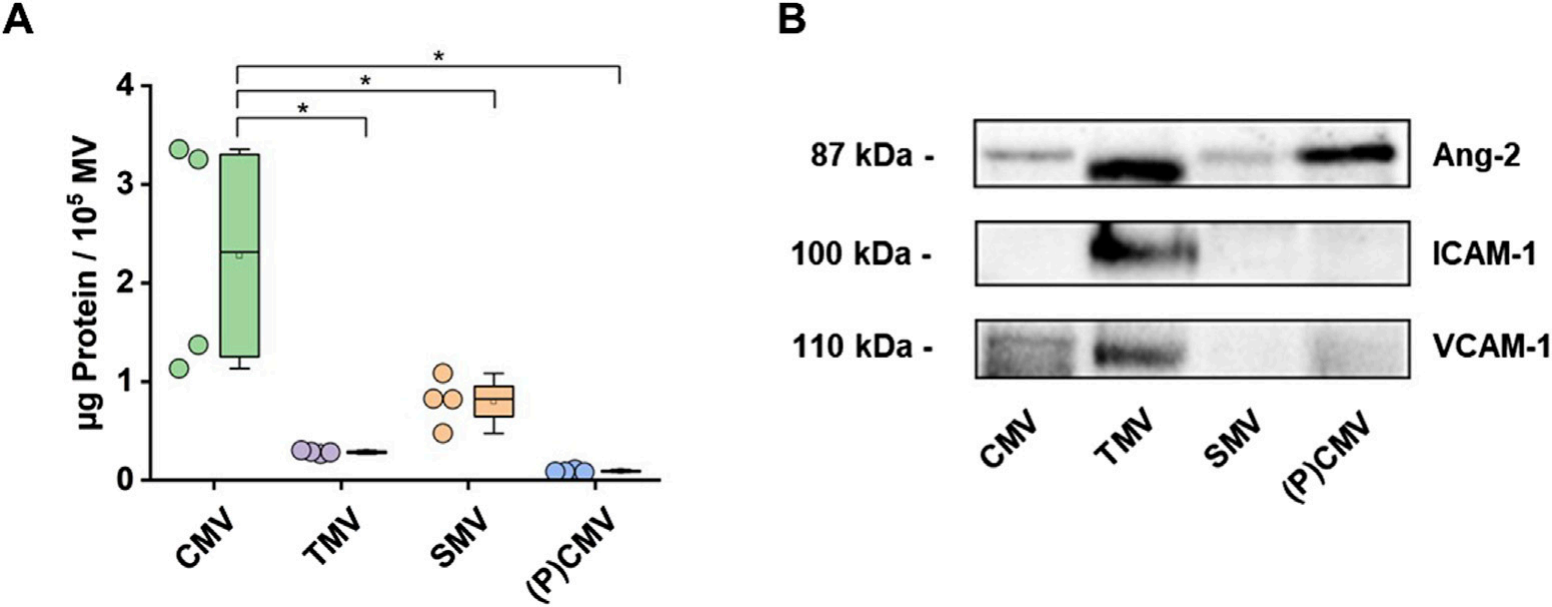
Characterization of MV. MV were isolated from the plasma of healthy volunteers (CMV), the supernatant of endothelial cell cultures stimulated with TNFα (100 ng/mL, 24 h; TMV), the plasma of septic patients (SMV), and a plasmapheresis procedure. **(A)** Protein concentration was determined using the Pierce™ 660 nm Protein Assay Kit. Data are shown as scatter dots and box plots indicating IQR range, mean, median, and whiskers extended to 1.5 IQR (n = 4 measurements). **(B)** MV preparations were analyzed by Western blot for Angiopoietin-2 (Ang-2), Intercellular Adhesion Molecule 1 (ICAM-1), and Vascular Cell Adhesion Molecule 1 (VCAM-1) protein content. Western blot images showing representative blots from two experiments.

The protein concentration of MV lysates was determined using the Pierce 660 nm Protein Assay Kit (#22662, Thermo Fisher Scientific, Waltham, USA) according to the manufacturer’s protocol, as described (Figure 3A) [20]. Standards, samples, and blanks were loaded into 96-well culture plates and combined with the provided protein reagent enhanced with the Ionic Detergent Compatible Reagent (IDCR, #22663, Thermo Fisher Scientific). Following a five-minute incubation at room temperature, absorbance was measured at 660 nm using an iMark microplate reader (Bio-Rad Laboratories, Hercules, USA). Protein concentrations were calculated by interpolation from the standard curve. Subsequently, 40 µg of total protein from each sample was loaded for SDS-PAGE and Western blot analysis.

#### SDS-PAGE Western blotting

MVs preparations were lysed using RIPA buffer supplemented with freshly prepared Pierce Protease and Phosphatase Inhibitor tablets (#A32959, Thermo Fisher Scientific) as described [20]. The lysates were vortexed and incubated on ice for 1 min to ensure complete EV disruption, followed by centrifugation at 12,000 × g for 30 min at 4°C. Supernatants were collected and combined with 5× Laemmli buffer, then heated at 95°C for 5 min to denature proteins and reduce disulfide bonds. Proteins were separated on a 10% SDS-PAGE gel and subsequently transferred onto a 0.45-μm PVDF membrane. To minimize nonspecific antibody binding, membranes were blocked with 1% bovine serum albumin (BSA) in TBS-T (Tris-buffered saline with 0.1% Tween-20) for 45 min at room temperature. Membranes were then incubated overnight at 4°C with primary antibodies. Primary antibodies were purchased from Santa Cruz Biotechnology, Inc. (Dallas, USA): Angiopoietin 2 (Ang-2; #sc-74403), ICAM-1 (#sc-107), and VCAM-1 (E10; #sc-13160). Anti-mouse secondary HRP-conjugated antibody (m-IgG1 BP-HRP; #sc-525408) was purchased from Santa Cruz Biotechnology, Inc. After washing with TBS-T to remove unbound antibodies, membranes were incubated with HRP-conjugated secondary antibodies for 1 h at room temperature. Following a final series of washes with TBS-T, protein bands were detected using enhanced chemiluminescence (ECL) reagents and imaged with the Chemi-Doc XRS+ system (Bio-Rad Laboratories, Figure 3B).

#### Cardiomyocyte treatment with MV

Human iPSC-CM were incubated with MV or PBS buffer (control) and experiments were then conducted. To assess different time points and concentrations, TMV (TNF-α-stimulated) and CMV (from unstimulated endothelial cells) were used. Initially 10^6^ endothelial MV/mL were added for 1, 3, 6, 16, and 24 h independently to investigate the time course of MV effects. Then, 3, 6, and 10 × 10^6^ endothelial MV/mL were added for 6 and 16 h to analyze the effect of different MV concentrations. Finally, SMV (sepsis patients’ MV) and (P)CMV were used and 10^7^ plasma MV/mL from different patients were added separately for 16 h.

### Functional analysis

#### Calcium transient acquisition

Spontaneous Ca^2+^ transients were measured using the ratiometric Ca^2+^-sensitive fluorescent indicator fura-2. Human iPSC-CM were loaded with 0.75 μM fura-2-AM (F1221, Thermo Fisher Scientific) diluted in Tyrode’s solution (pH 7.4, solution composition (in mM): NaCl 140, KCl 5.4, CaCl_2_ 1.8, MgCl_2_ 1.1, HEPES 10, and glucose 10) for 20 min at room temperature in the dark. After 10 min of de-esterification, spontaneous Ca^2+^ release activity was recorded using the IonOptix system (IonOptix, Dublin, Ireland). Cells were exposed to light emitted by a Xenon lamp passing through rapidly switching filters of 340 nm and 380 nm excitation wavelengths. The ratio of Ca^2+^-bound (numerator) and unbound (denominator) fura-2 was determined and fluorescence emission was collected at 510 nm. Data were collected using the IonWizard software and are presented as fura-2 ratio (F_340_/F_380_).

#### Calcium transient analysis

**FIGURE 4:**
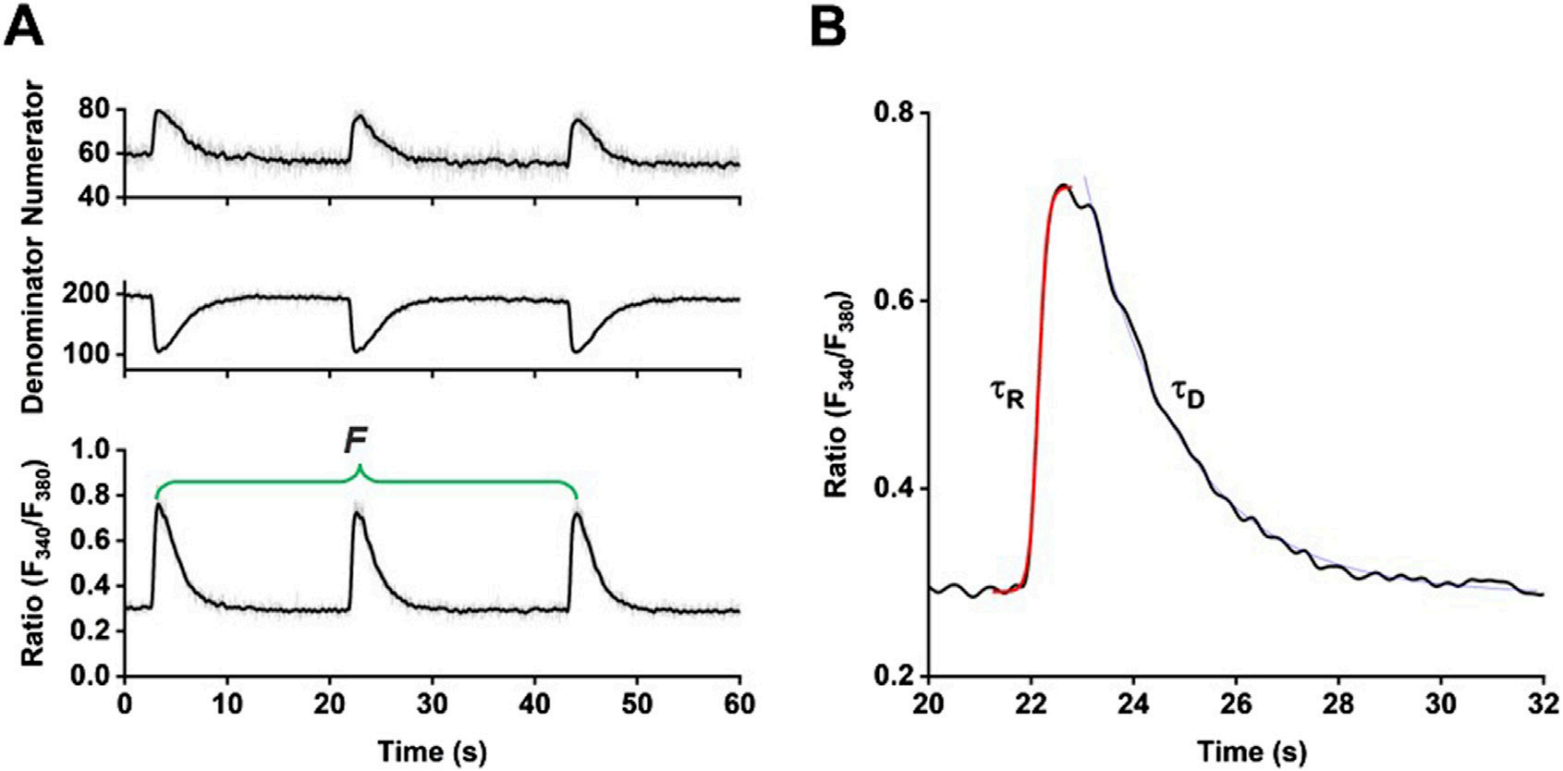
Functional analysis of spontaneous Ca^2+^ transients. **(A)** Sample traces of Ca^2+^ transients recorded using the ratiometric Ca^2+^-sensitive indicator fura-2. **(B)** Enlargement of one Ca^2+^ transient and indication of the time constants τ_R_ and τ_D_ derived from sigmoidal and exponential decay fitting procedures, respectively.

For functional evaluation of spontaneous activity only regular beating hiPSC-CM were used. Three representative Ca^2+^ transients were analyzed at a steady state. To evaluate changes in Ca^2+^ release and reuptake activity in the different experimental groups, parameters were analyzed by assessing the time constants of Ca^2+^ rise (τ_R_) and Ca^2+^ decay (τ_D_) and the beating frequency using OriginPro^®^ (Origin Lab Corporations, Northampton, MA, United States). τ_R_ was fitted with a sigmoidal function, whereas τ_D_ was fitted with an exponential decay function (Figure 4). Exclusion criteria for data analysis were set as Ca^2+^ peak below 0.4 F_340_/F_380_, Ca^2+^ baseline above 0.6 F_340_/F_380_, Time-To-Peak (TTP) above 2000 ms, τ_D_ above 4000 ms, and abnormal transient shapes.

### Data and statistical analysis

Experiments were repeated in three rounds with three different stem cell passages for the HPMEC experiments. “n” denotes the number of individually analyzed cells (one data point), whereas “N” designates the analysis number of GBD, equivalent to three different hiPSC-CM passages for endothelial MV experiments and 16 different patients for the plasma MV experiment. Graphic design and statistical analysis were performed using OriginPro^®^ (OriginLab software) and MV-count calculations using Excel. Normality was determined by a Shapiro-Wilk test and homoscedasticity by the Levens test. To evaluate the effect that MV may have on the Ca^2+^ transient parameters of hiPSC-CM based on the proposed hypothesis, the non-parametric Kruskal-Wallis test was conducted to test for differences between TMV or SMV-, CMV-, and PBS-treated cardiomyocytes, followed by the Dunns post-hoc-test with a Bonferroni alpha error adjustment to compare two conditions to each other. Most of the data are illustrated as box plots representing each data point (dots), mean (black square), median (centre line), 25th −75th quantiles (box edges), and standard deviation (SD, whiskers). Data in the results are presented as mean ± SD. Statistical significance is indicated by * for p < 0.05.

## Results

Stimulation of HPMECs with TNFα increased the concentration of MV in the supernatant by a factor of 5.8 (difference between means [stimulated vs. unstimulated] ± SEM: 79,097 ± 5 455; *p* < 0.0001; *n* = 5). The difference between TMV and CMV was 6.4-fold (difference between means [TMV–CMV] ± SEM: 251,676 ± 16,404; *p* < 0.0001; *n* = 4). Plasma from septic patients contained fewer MVs per µL than the supernatant from TNFα-stimulated HPMECs (difference between means [SMV–TMV] ± SEM: −169 034 ± 26,046; *p* < 0.001; *n* = 4). Protein content, on the other hand, was highest in CMV (Figure 3A). All MV preparations analyzed in this study consistently contained angiopoietin-2 (Ang-2) as part of their protein cargo, as confirmed by Western blot (Figure 3B; Supplementary Figure S3). MV from TNFα-stimulated HPMEC (TMV) also contained Intercellular Adhesion Molecule 1 (ICAM-1), whereas MV isolated from unstimulated HPMEC (CMV), septic patients, or healthy volunteers did not. Vascular Cell Adhesion Molecule 1 (VCAM-1) was found in all MV preparations except in MV from septic patients.

After differentiation and metabolic selection of hiPSC-CM, cells were incubated either with MV isolated from unstimulated HPMEC (CMV) or from TNFα-stimulated HPMEC (TMV). PBS-treated cells served as MV-negative control. In time-course experiments, different incubation times of MV on hiPSC-CM were evaluated, while in concentration-dependent experiments, different concentrations of MV were tested at two different time points. For the functional experiments, hiPSC-CM were loaded with fura-2 and Ca^2+^ transients were recorded over time. Since the rise and decay of the Ca^2+^ transients are sensitively regulated processes during the mechanism of excitation-contraction coupling, these parameters were evaluated, together with the spontaneous frequency of these transients, representative of the beating frequency. Details of the strategy to analyze Ca^2+^ transients are summarized in Figure 4 and indicate the time constants of τ_R_ and τ_D_ derived from curve fitting as well as the frequency (F).

**FIGURE 5:**
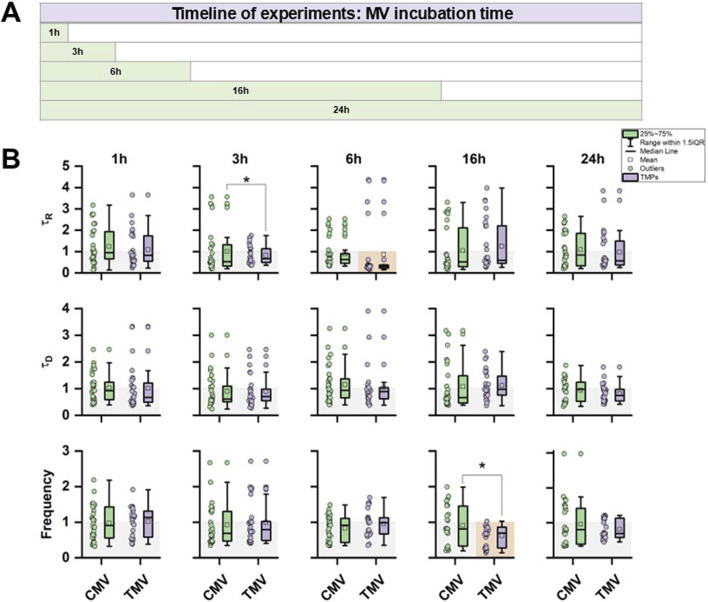
Time course of the effect of control (CMV) and TNFα-induced MV (TMV) from HPMEC on hiPSC-CM. **(A)** Protocol of the time course experiments indicating data collection after 1, 3, 6, 16, or 24 h of incubation. **(B)** Statistical summary of τ_R_, τ_D_, and *F* from hiPSC-CM Ca^2+^ transients from CMV and TMV treatment. Data are normalized to PBS control. Significances between CMV and TMV are indicated as * for P < 0.05; significances to PBS control are indicated as orange box. Experiments were repeated on three different hiPSC passages and differentiations. Each data point summarizes the average of 3 Ca^2+^ transients per cell, with a group size of 30 cells total per group.

In the first set of functional experiments, hiPSC-CM were incubated with 1 × 10^6^ MV derived from either TNFα-stimulated or control HPMEC; PBS treatment served as MV-free control. The protocol of the time course experiment is outlined in Figure 5A. Ca^2+^ transients were evaluated at five different time points. Figure 5B shows the summary of the statistical evaluation of τ_R_, τ_D_, and *F* derived from the Ca^2+^ transients. Data were normalized to PBS control (raw data are summarized in Supplementary Figure S1). After 3 h of incubation, TMV-treated hiPSC-CM showed a faster rise in the Ca^2+^ transients compared to CMV-treated cells with reduced data variability. After 6 h of incubation, TMV-treated cells had even shorter τ_R_ than PBS controls (indicated by the orange background). While under these conditions, no difference was detected in the decay kinetics of the Ca^2+^ transients, and the frequency was significantly lower after 16 h of TMV-treatment compared to CMV and PBS (two-sample t-test: P = 0.0224).

**FIGURE 6:**
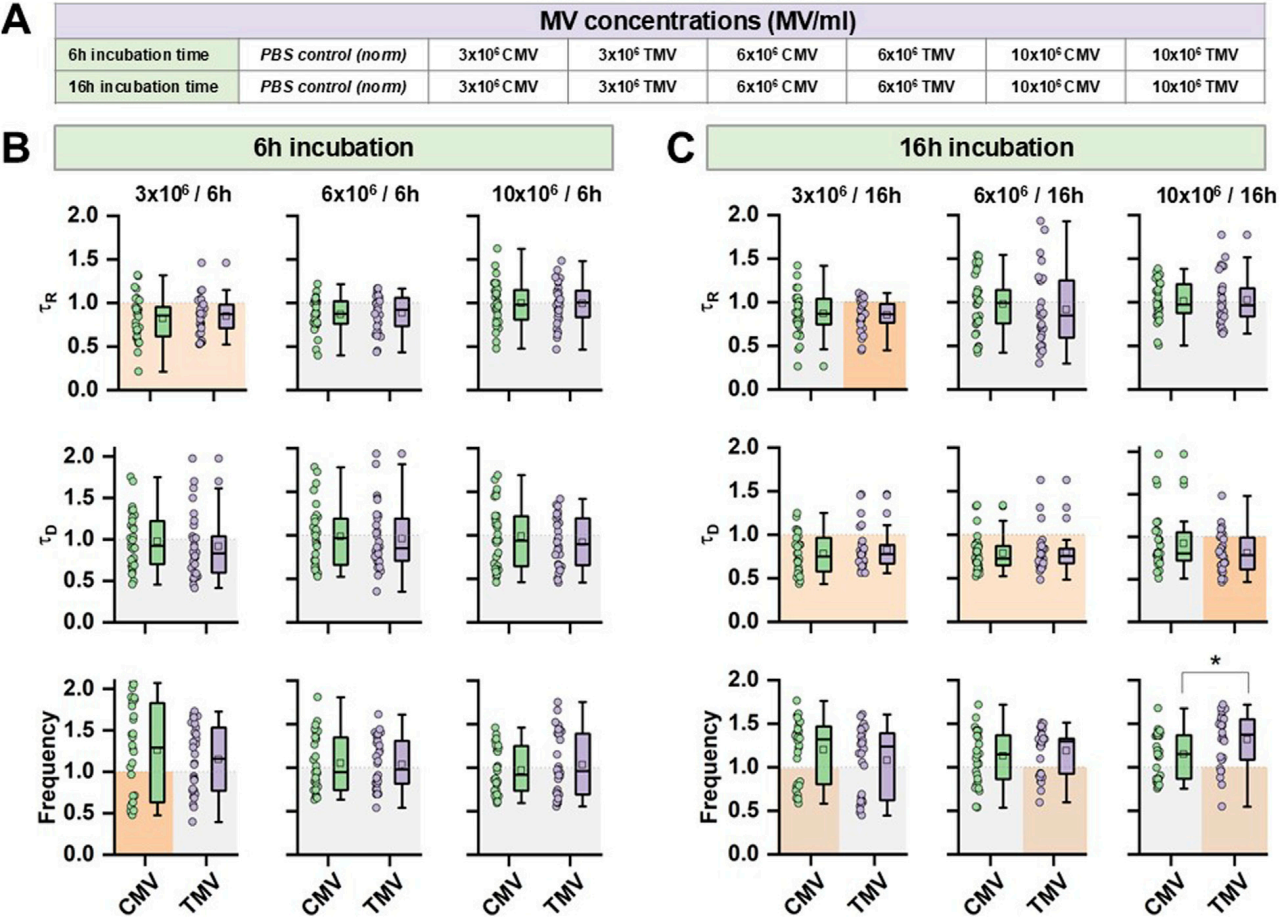
Concentration-dependence of the effect of CMV and TMV from HPMEC on hiPSC-CM relative to PBS. **(A)** Protocol of the dose-dependent experiments indicating data collection after 6 or 16 h of incubation. **(B,C)** Statistical summary of τ_R_, τ_D_, and *F* from hiPSC-CM Ca^2+^ transients from CMV and TMV treatment at two different time points, respectively. Data are normalized to PBS control. Significances between CMV and TMV are indicated as * for P < 0.05; significances to PBS control are indicated as orange box. Experiments were repeated on three different hiPSC passages and differentiations. Each data point summarizes the average of three Ca^2+^ transients per cell, with a group size of 30 cells total per group.

To investigate the influence of different MV concentrations, hiPSC-CM were incubated with three distinct amounts of CMV or TMV, and the effect on Ca^2+^ transient kinetics were assessed at two different time points, namely after 6 and 16 h of incubation. Figure 6A summarizes the experimental strategy for the test of 3 × 10^6^, 6 × 10^6^, or 10 × 10^6^ MV on Ca^2+^ transient function. The data for the statistical evaluation of τ_R_, τ_D_, and *F* derived from Ca^2+^ transients are given in Figure 6B (raw data are summarized in Supplementary Figure S2). Compared to PBS control, CMV- and TMV-treated hiPSC-CM had faster rises of Ca^2+^ transients at 3 × 10^6^ MV at 6 (CMV and TMV) and 16 h (TMV only) of incubation. However, this effect was not present at higher MV concentrations. Longer incubation times had a significant effect on the decay of Ca^2+^ transients. Compared to PBS control after 16 h, CMV- and TMV-treated cells had a smaller τ_D_, indicating faster return of [Ca^2+^]_i_ to baseline levels. Moreover, the frequency of spontaneous Ca^2+^ transients was also modified relative to PBS control, with a shift toward higher frequencies. At 10 × 10^6^ MV and 16 h, TMV induced a significantly faster beating rate compared to CMV treatment.

**FIGURE 7:**
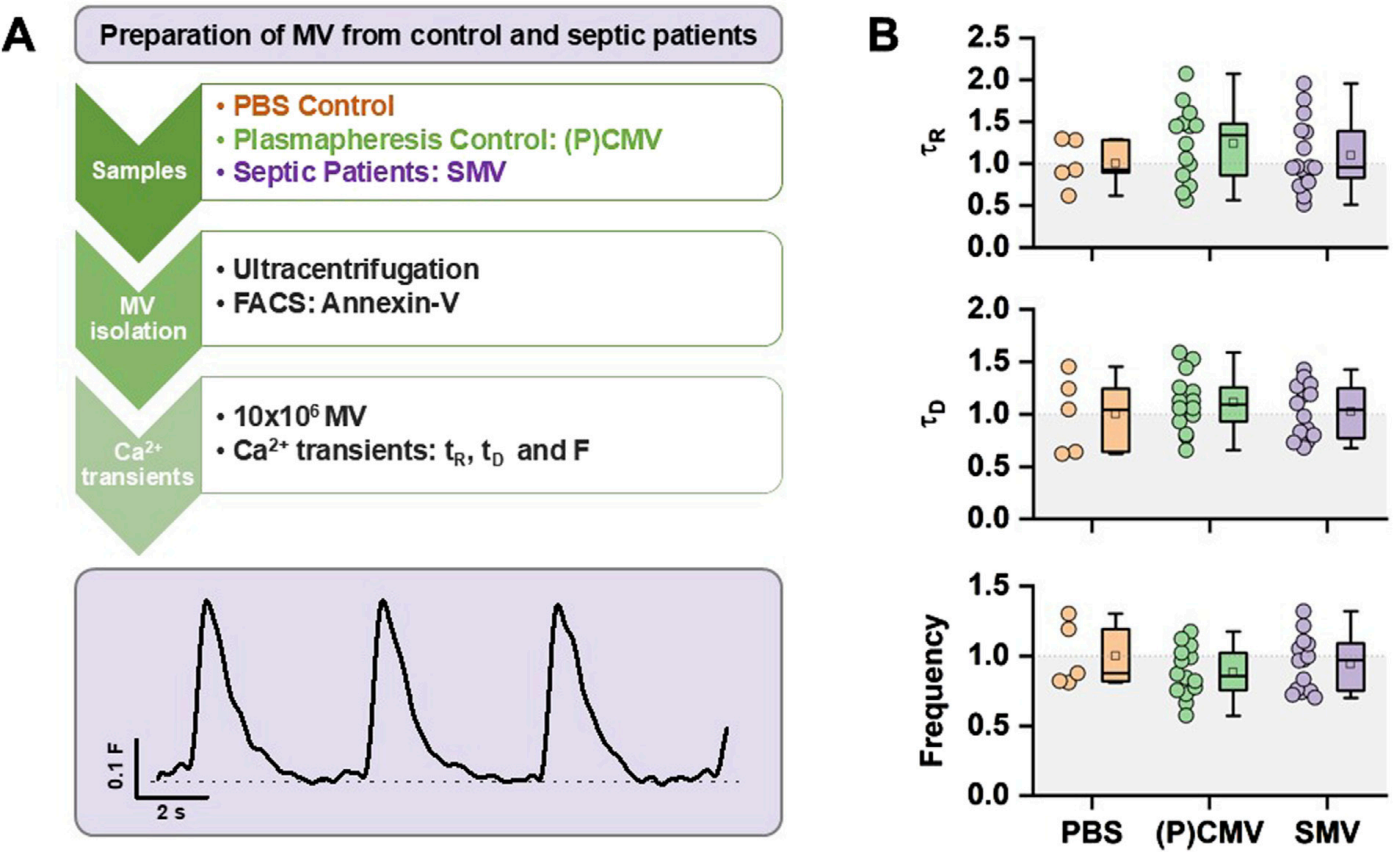
Effect of septic MV from patients on hiPSC-CM. **(A)** Strategy of experimental setup. hiPSC-CM were incubated for 16 h with MV before analysis of Ca^2+^ transients. **(B)** Statistical summary of τ_R_, τ_D_, and *F* from hiPSC-CM Ca^2+^ transients from PBS, (P)CMV, and SMV treatment. Data are normalized to PBS control. Experiments were carried out from one hiPSC passage and differentiation. Each data point summarizes the average of 3 Ca^2+^ transients per cell, with a group size of five (PBS), 14 ((P)CMV)), and 16 (SMV) cells total per group.

In the last set of experiments, the effect of MV isolated from sepsis patients was studied. Septic patients were 66.4 ± 9.6 years of age and had a median SOFA score of 11 (6.8; 12). The data are summarized in Table 1. Figure 7A demonstrates the experimental flow. In this case, MV were isolated from plasmapheresis samples as negative control ((P)CMV) and from septic patients (SMV). hiPSC-CM were incubated with 10 × 10^6^ MV for 16 h. Data were again normalized to PBS controls. The statistical evaluation of τ_R_, τ_D_, and *F* measured from Ca^2+^ transients did not reveal any significant differences after treatment of the cells with (P)CMV or SMV relative to control (Figure 7B).

**TABLE 1:** The baseline characteristics of the patients.

Variable		Patients
n		16
Age, years		66.4 ± 9.6
Male sex, *n*		11
Sepsis focus	Lung	1
	Abdominal	11
	Urinary	1
Use of mechanical ventilation, *n* (%)		75
Duration of mechanical ventilation, days		4.4 ± 6.3
SOFA score D1		10.0 ± 3.4
APACHE II score D1		27.3 ± 8.0
ICU length of stay, days		5.1 ± 8.0
Hospital length of stay, days		20.7 ± 16.7
28-day hospital mortality, n (%)		12.5
MAP (mmHg)		78.2 ± 15.4
Lactate (mmol/L)		2.3 ± 1.3
PCT, ng/mL		10.0 ± 16.8
CRP, mg/L?		227.2 ± 88.5
WBC,10^12^/L		13.7 ± 9.0

*SOFA* sequential organ failure assessment score, *APACHE* acute physiology and chronic health evaluation, *ICU* intensive care unit, *MAP* mean arterial pressure, *PCT* procalcitonin, *WBC* white blood cells, *CRP* c-reactive protein. Data are presented as mean ± SD or percentage (%).

## Discussion

Our study aimed to investigate the potential impact of MV on the contractility of hiPSC-CM in a novel experimental model for septic cardiomyopathy. MV can contribute to apoptosis and inflammation of endothelial cells, which can cause the endothelial barrier to become leaky [20]. Moreover, as a result of the systemic inflammatory response associated with sepsis, impaired endothelial function and myocardial dysfunction might arise, potentially mediated by MV.

Here, we focused on a specific subset of MV derived from HPMEC, a primary endothelial cell line that closely reflects cell function of the microvasculature. The study analyzed the effect of TNFα-derived TMV and SMV from septic patients on hiPSC-CM. As a functional readout, the temporal kinetics of Ca^2+^ transients were chosen. Ca^2+^-induced Ca^2+^ release is the primary mechanism that bridges cardiomyocyte membrane activation (the action potential) to cell contraction [21, 22]. Therefore, influences that affect the contractility of cardiomyocytes can be reflected in changes in the release properties of this important intracellular messenger, Ca^2+^. Particular attention was paid to the rise and decay kinetics of the Ca^2+^ transients and to the beating frequency of the cells. Despite isolated significant results in the presented data sets, the experiments revealed that TMV and SMV did not induce consistent responses in hiPSC-CM. These experiments demonstrate that MV have the potential to influence Ca^2+^ transient kinetics of hiPSC-CM, especially regarding the frequency of the Ca^2+^ release events. The upregulation of pro-apoptotic molecules and intercellular adhesion molecules were described in other cells after incubation with TMV [21]. A similar effect might lie behind our findings. However, the high data variability, which is intrinsic to hiPSC-CM function, may mask stronger effects and impede the identification of significances.

Nevertheless, even subtle alterations in Ca^2+^ transient dynamics, such as changes in amplitude, rise time, or decay kinetics, can significantly impact EC-coupling in cardiomyocytes. Even minor changes may translate into impaired systolic contractility, delayed relaxation, and reduced Ca^2+^ reuptake efficiency, all of which are hallmarks of septic cardiomyopathy.

Moreover, disturbances in Ca^2+^ handling can promote arrhythmogenic conditions by destabilizing the membrane potential and increasing the susceptibility to afterdepolarizations. In the context of sepsis, where mitochondrial dysfunction, oxidative stress, and inflammatory mediators are abundant, such minor impairments may become functionally significant due to reduced cardiac reserve.

Another possible explanation for the high data variability could be attributed to the experimental limitations inherent in our model. While our *in vitro* model attempted to replicate constant physiological conditions, it is crucial to acknowledge that *in vitro* systems may not fully recapitulate the complex microenvironment present *in vivo*. Factors such as cell culture conditions, the purity and composition of MV preparation, and the specific characteristics of hiPSC-CM may influence the outcome of these experiments. Nevertheless, the variability in the effects of MV in the Ca^2+^ transient analyses likely reflect genuine biological heterogeneity, which is consistent with previously reported findings [23, 24].

Furthermore, the heterogeneity of the different MV populations and their diverse cargo contents add another layer of complexity to this study. Although MV used in these experiments were derived from one specific cell type, the HPMEC, future investigations may benefit from exploring a broader range of MV populations to elucidate potential functional differences. This is particularly relevant given that we observed significant differences in the number, protein content, and cargo between SMVs and TMVs. The inflammatory environment in septic patients is highly complex, involving a combination of pro-inflammatory cytokines associated with the systemic inflammatory response characteristic of sepsis (e.g., IL-1β, IL-6, TNF-α, and interferon-γ). For future experiments, it may be beneficial to use either a defined mix of these cytokines or plasma from patients in the acute phase of sepsis to better replicate the *in vivo* conditions.

Exosomes from patients with septic shock have been described to convey miRNAs and mRNAs related to pathogenic pathways, including inflammatory response, oxidative stress, and cell cycle regulation [15]. Therefore, exosomes may represent a novel mechanism for intercellular communication during sepsis. Since MV could originate from various cells affected by the pathophysiology of sepsis, their origin, activation, and the immunological state of the parent cell most likely influence the content and effects of MV. However, there is currently no clear evidence regarding quantity, cargo, and time course of MV during sepsis progression in patients. Hence, we based the concentrations used in our *in vitro* experiments (3–10 × 10^6^ MV/mL) on prior *in vitro* studies that demonstrated functional effects within similar concentration windows.

Additionally, the dynamic nature of intercellular communication mediated by MV warrants consideration. While our experimental setup allowed for the direct exposure of cardiomyocytes to MV, it is possible that the observed effects are transient or context-dependent.

Here, we selected the 6-h and 16-h timepoints based on a combination of practical usability, biological relevance, and protein expression kinetics in hiPSC-CM. Regarding usability and cellular viability, timepoints beyond 24 h were associated with increased cell stress and declining viability in pilot experiments, limiting the interpretability of downstream readouts. The 6–16 h window allowed us to capture early and intermediate cellular responses while preserving cell health and morphology. With respect to protein expression kinetics, our primary endpoints included markers of inflammatory response and functional proteins such as contractile elements and metabolic regulators. Previous studies, as well as our own kinetics profiling, suggest that differential protein expression in hiPSC-CM in response to inflammatory stimuli is detectable between 6- and 16-h post-exposure. The 6-h timepoint captures early signaling events and transcriptional activation, while the 16-h timepoint reflects post-transcriptional and translational outcomes relevant for phenotypic changes. While the dynamics of circulating SMV concentrations in patients vary based on the severity and phase of sepsis, elevated levels are typically sustained over several hours to days during the acute phase. Thus, exposing hiPSC-CM to SMVs for 6 and 16 h reflects clinically plausible exposure durations within this window, allowing us to model both acute-onset and sustained exposure scenarios relevant to septic cardiac dysfunction.

Long-term studies tracking the fate of MV and their effects on cardiomyocyte function over time could provide valuable insights into the temporal dynamics of MV-mediated signaling. A key limitation of this study is the exclusive use of hiPSC-derived cardiomyocytes (hiPSC-CMs), which, despite their relevance as a human-based model, do not fully recapitulate the structural complexity, cellular heterogeneity, and long-term remodeling responses of native cardiac tissue. In particular, the absence of multicellular interactions, vascularization, and tissue-level organization may limit the translational applicability of our findings. Future studies will incorporate more complex model systems such as cardiac organoids, engineered heart tissues, or *in vivo* models to validate and extend these results under more physiologically relevant conditions. In addition, longitudinal studies will be essential to assess the durability and adaptive nature of the observed responses over time.

Our findings that Ang-2 is present in all MV preparations analyzed confirm their endothelial origin. Given the established role of Ang-2 in endothelial activation and vascular permeability, its presence in MV could contribute to the propagation of endothelial dysfunction in inflammatory or septic conditions. As Ang-2 is known to destabilize endothelial junctions, promote vascular leakage, and amplify inflammation, it may indirectly impair cardiomyocyte oxygenation, survival, and function via endothelial dysfunction. In line with the findings of Chatterjee et al, we also found TNFα to induce the production of MVs that express markers of cell injury or activation in endothelial cells [25]. We would have also expected the MV from septic patients to express ICAM-1 and VCAM-1 as these are typical for proinflammatory signaling, immune cell recruitment, and inflammation. Although ICAM-1 and VCAM-1 are well-known markers of endothelial activation and play central roles in leukocyte adhesion and inflammation, their absence in MV derived from septic patients may reflect a selective packaging mechanism that favors the inclusion of intracellular or membrane-associated proteins involved in vesicle formation, signaling, or stress response—rather than classical surface adhesion molecules.

Additionally, shedding of MV may occur from endothelial regions or cellular compartments where ICAM-1 and VCAM-1 are either not highly expressed or are retained on the parent cell surface to fulfill their adhesion functions. It is also possible that under the conditions of severe systemic inflammation, proteolytic cleavage or internalization of these molecules limits their availability for incorporation into vesicles.

Finally, MV cargo composition may be influenced by disease stage, cytokine milieu, or oxidative stress, leading to altered protein sorting that deprioritizes adhesion molecules in favor of other proinflammatory mediators (e.g., cytokines, danger signals, or coagulation-related proteins).

Further identification of the content and the effect of MV on the heart cells will provide important information for the use of MV for diagnostic and therapeutic purposes. Both the content and the membranes can be engineered independently and thus be used for different purposes and applications [9].

In conclusion, while our study did not reveal any consistent impact of MV on cardiomyocyte contractility under most of the conditions tested, it is essential to recognize the complexity of intercellular communication mediated by MV. Further investigations incorporating refined experimental models, diverse MV populations, and longitudinal analyses are warranted to fully elucidate the role of MV in modulating cardiac function, especially during sepsis. These efforts will contribute to a deeper understanding of MV biology and may uncover novel therapeutic avenues for septic cardiomyopathy.

## Data Availability

The original contributions presented in the study are included in the article/Supplementary Material, further inquiries can be directed to the corresponding author.

## References

[1] Parker MM Shelhamer JH Bacharach SL Green MV Natanson C Frederick TM Profound but reversible myocardial depression in patients with septic shock. Ann Intern Med (1984) 100(4):483–90. 10.7326/0003-4819-100-4-483 6703540

[2] Ehrman RR Sullivan AN Favot MJ Sherwin RL Reynolds CA Abidov A Pathophysiology, echocardiographic evaluation, biomarker findings, and prognostic implications of septic cardiomyopathy: a review of the literature. Crit Care (2018) 22(1):112. 10.1186/s13054-018-2043-8 29724231 PMC5934857

[3] Lin H Wang W Lee M Meng Q Ren H. Current status of septic cardiomyopathy: basic science and clinical progress. Front Pharmacol (2020) 11:210. 10.3389/fphar.2020.00210 32194424 PMC7062914

[4] Reid VL Webster NR. Role of microparticles in sepsis. Br J Anaesth (2012) 109(4):503–13. 10.1093/bja/aes321 22952169

[5] Terrasini N Lionetti V. Exosomes in critical illness. Crit Care Med (2017) 45(6):1054–60. 10.1097/ccm.0000000000002328 28328650

[6] Weber B Henrich D Hildebrand F Marzi I Leppik L. The roles of extracellular vesicles in sepsis and systemic inflammatory response syndrome. Shock (2023) 59(2):161–72. 10.1097/shk.0000000000002010 36730865 PMC9940838

[7] Hashemian SM Pourhanifeh MH Fadaei S Velayati AA Mirzaei H Hamblin MR. Non-coding RNAs and exosomes: their role in the pathogenesis of sepsis. Mol Ther Nucleic Acids (2020) 21:51–74. 10.1016/j.omtn.2020.05.012 32506014 PMC7272511

[8] Ginini L Billan S Fridman E Gil Z. Insight into extracellular vesicle-cell communication: from cell recognition to intracellular fate. Cells (2022) 11(9):1375. 10.3390/cells11091375 35563681 PMC9101098

[9] Kao CY Papoutsakis ET. Extracellular vesicles: exosomes, microparticles, their parts, and their targets to enable their biomanufacturing and clinical applications. Curr Opin Biotechnol (2019) 60:89–98. 10.1016/j.copbio.2019.01.005 30851486

[10] Azevedo LC Janiszewski M Pontieri V Pedro MA Bassi E Tucci PJF Platelet-derived exosomes from septic shock patients induce myocardial dysfunction. Crit Care (2007) 11(6):R120. 10.1186/cc6176 17996049 PMC2246209

[11] Mortaza S Martinez MC Baron-Menguy C Burban M de la Bourdonnaye M Fizanne L Detrimental hemodynamic and inflammatory effects of microparticles originating from septic rats. Crit Care Med (2009) 37(6):2045–50. 10.1097/ccm.0b013e3181a00629 19384196

[12] Zhang Q Shang M Zhang M Wang Y Chen Y Wu Y Microvesicles derived from hypoxia/reoxygenation-treated human umbilical vein endothelial cells promote apoptosis and oxidative stress in H9c2 cardiomyocytes. BMC Cell Biol (2016) 17(1):25. 10.1186/s12860-016-0100-1 27338159 PMC4919832

[13] Zhang S Yin Y Li C Zhao Y Wang Q Zhang X. PAK4 suppresses TNF-induced release of endothelial microparticles in HUVECs cells. Aging (Albany NY) (2020) 12(13):12740–9. 10.18632/aging.103173 32657762 PMC7377857

[14] Liu Y Zhang R Qu H Wu J Li L Tang Y. Endothelial microparticles activate endothelial cells to facilitate the inflammatory response. Mol Med Rep (2017) 15(3):1291–6. 10.3892/mmr.2017.6113 28098900

[15] Real JM Ferreira LRP Esteves GH Koyama FC Dias MVS Bezerra-Neto JE Exosomes from patients with septic shock convey miRNAs related to inflammation and cell cycle regulation: new signaling pathways in sepsis? Crit Care (2018) 22(1):68. 10.1186/s13054-018-2003-3 29540208 PMC5852953

[16] Ye R Lin Q Xiao W Mao L Zhang P Zhou L miR-150-5p in neutrophil-derived extracellular vesicles associated with sepsis-induced cardiomyopathy in septic patients. Cell Death Discov (2023) 9(1):19. 10.1038/s41420-023-01328-x 36681676 PMC9867758

[17] Rossler U Hennig AF Stelzer N Bose S Kopp J Søe K Efficient generation of osteoclasts from human induced pluripotent stem cells and functional investigations of lethal CLCN7-related osteopetrosis. J Bone Mineral Res (2021) 36(8):1621–35. 10.1002/jbmr.4322 33905594

[18] Zhao M Tang Y Zhou Y Zhang J. Deciphering role of Wnt signalling in cardiac mesoderm and cardiomyocyte differentiation from human iPSCs: four-dimensional control of Wnt pathway for hiPSC-CMs differentiation. Sci Rep (2019) 9(1):19389. 10.1038/s41598-019-55620-x 31852937 PMC6920374

[19] Singer M Deutschman CS Seymour CW Shankar-Hari M Annane D Bauer M The third international consensus definitions for sepsis and septic shock (Sepsis-3). JAMA (2016) 315(8):801–10. 10.1001/jama.2016.0287 26903338 PMC4968574

[20] Lajqi T Köstlin-Gille N Hillmer S Braun M Kranig SA Dietz S Gut microbiota-derived small extracellular vesicles endorse memory-like inflammatory responses in murine neutrophils. Biomedicines (2022) 10(2):442. 10.3390/biomedicines10020442 35203650 PMC8962420

[21] Lee SK Yang SH Kwon I Lee OH Heo JH. Role of tumour necrosis factor receptor-1 and nuclear factor-κB in production of TNF-α-induced pro-inflammatory microparticles in endothelial cells. Thromb Haemost (2014) 112(3):580–8. 10.1160/th13-11-0975 25008247

[22] Bers DM. Cardiac excitation-contraction coupling. Nature (2002) 415(6868):198–205. 10.1038/415198a 11805843

[23] Kermani F Mosqueira M Peters K Lemma ED Rapti K Grimm D Membrane remodelling triggers maturation of excitation-contraction coupling in 3D-shaped human-induced pluripotent stem cell-derived cardiomyocytes. Basic Res Cardiol (2023) 118(1):13. 10.1007/s00395-023-00984-5 36988697 PMC10060306

[24] Silbernagel N Körner A Balitzki J Jaggy M Bertels S Richter B Shaping the heart: structural and functional maturation of iPSC-cardiomyocytes in 3D-micro-scaffolds. Biomaterials (2020) 227:119551. 10.1016/j.biomaterials.2019.119551 31670034

[25] Chatterjee V Yang X Ma Y Cha B Meegan JE Wu M Endothelial microvesicles carrying Src-rich cargo impair adherens junction integrity and cytoskeleton homeostasis. Cardiovasc Res (2020) 116(8):1525–38. 10.1093/cvr/cvz238 31504252 PMC7314637

