# Supplementary material for: An approach to evaluate the effect of inflammatory microvesicles on Ca2+ handling in human-induced pluripotent stem cell-derived cardiomyocytes
Source: Exp Biol Med (Maywood). 2025 Aug 28;250:10461. doi: 10.3389/ebm.2025.10461 (PMC12422983; doi:10.3389/ebm.2025.10461)
Supplement: Supplementary file 1 [file DataSheet1.pdf]

Supplemental Information

An approach to evaluate the effect of inflammatory microvesicles on Ca^{2+} handling in human induced pluripotent stem cell-derived cardiomyocytes

Dania Fischer, Mishkaat Sha'sha'a, Judith Schenz, Aycan Tayan, Christina Mertens, Sebastian O Decker, Nadia Gallenstein, Maximilian Dietrich, Trim Lajqi, Anna Hafner, Markus A Weigand, Nina D Ullrich*

Microvesicles (MV) from septic patients may influence systemic hemodynamics and cardiac function. In this study, TNFα-induced endothelial MV (TMV) significantly decreased the beating frequency of human iPSC-derived cardiomyocytes after 16 hours of incubation, while MV from septic patients (SMV) did not show significant effects on Ca^{2+} transients. Although MV from control and TNFα-treated endothelial cells affected Ca^{2+} handling and spontaneous activity, the results were inconsistent, indicating a need for further refinement of experimental conditions to clarify the interaction between endothelium-derived MV and cardiomyocytes.

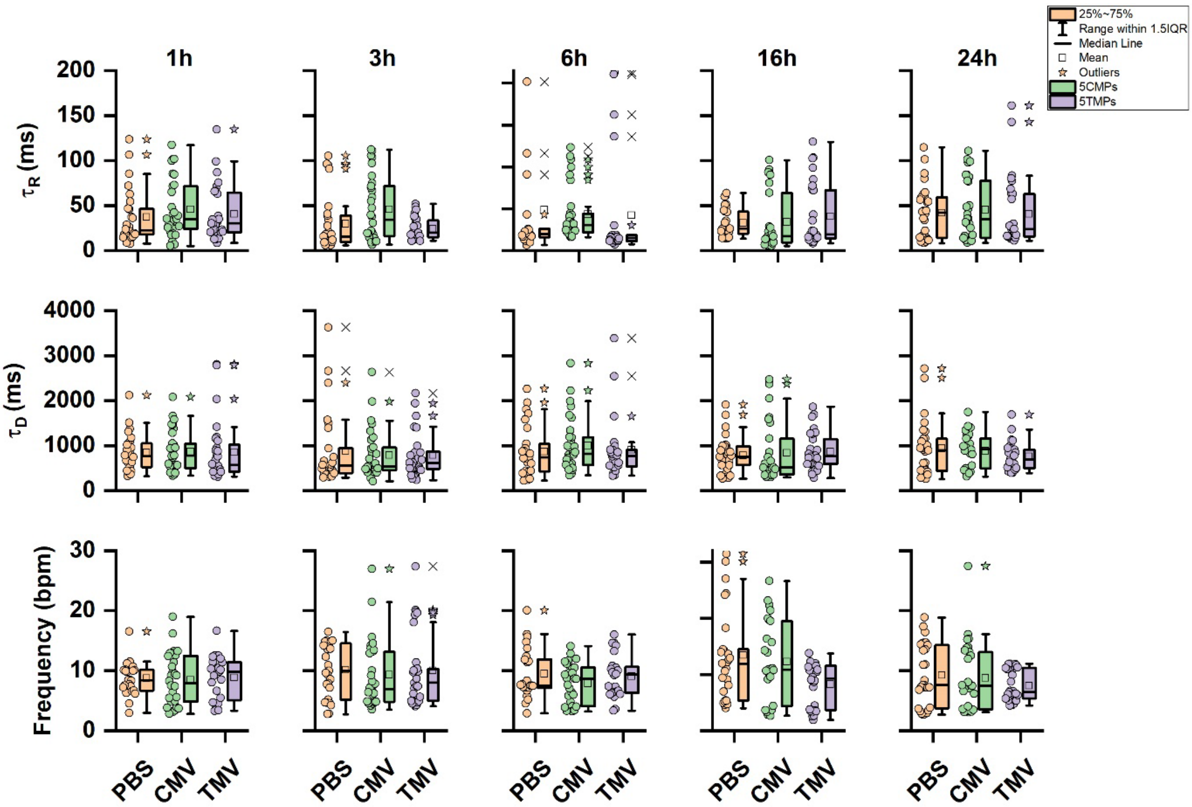

Supplemental Figure 1: Time course of the effect of control (CMV) and TNFα-induced MV (TMV) from HPMEC on hiPSC-CM. Statistical summary of τ_R, τ_D and F from hiPSC-CM Ca^{2+} transients from CMV and TMV treatment. Data show raw values for PBS control, CMV- and TMV-treated cells. Experiments were repeated on 3 different hiPSC passages and differentiations. Each data point summarizes the average of 3 Ca^{2+} transients per cell, with a group size of 30 cells total per group.

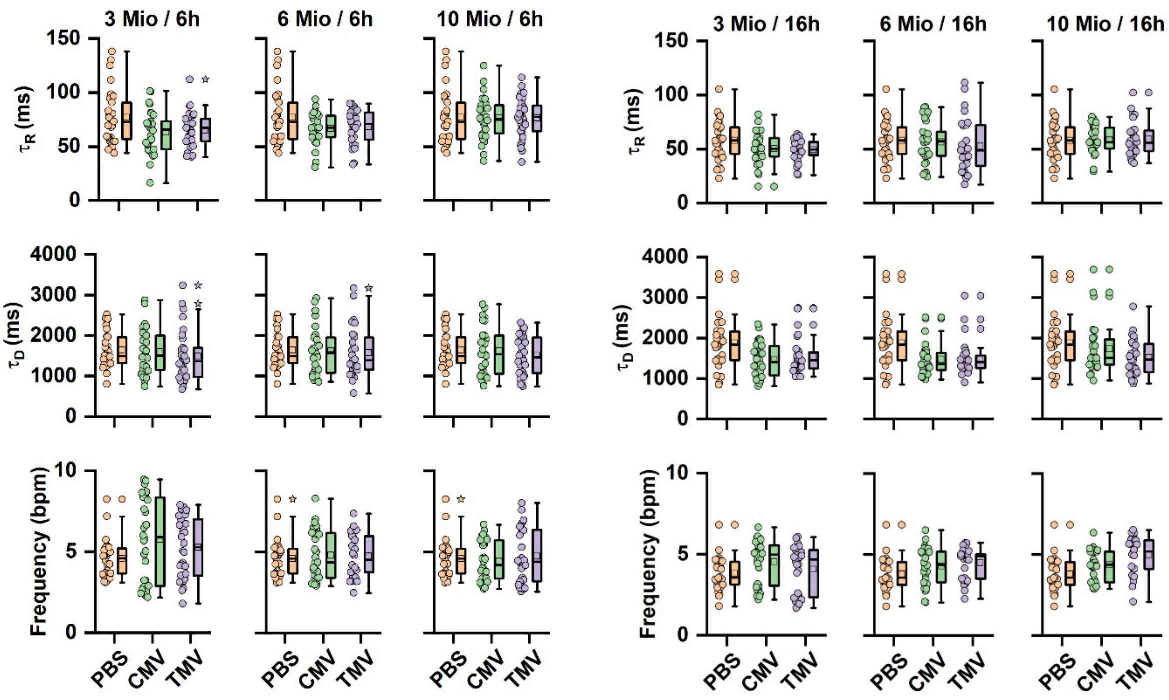

Supplemental Figure 2: Concentration-dependence of the effect of CMV and TMV from HPMEC on hiPSC-CM relative to PBS. Statistical summary of τ_R, τ_D and *F* from hiPSC-CM Ca^{2+} transients from CMV and TMV treatment. Data show raw values for PBS control, CMV- and TMV-treated cells. Experiments were repeated on 3 different hiPSC passages and differentiations. Each data point summarizes the average of 3 Ca^{2+} transients per cell, with a group size of 30 cells total per group.

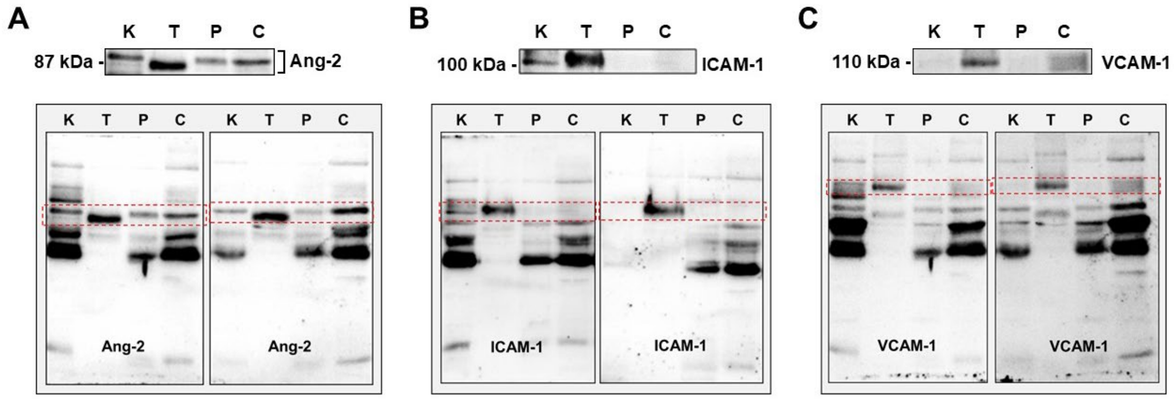

Supplemental Figure 3: Original Western blot images of MV preparations showing data from two individual experiments each. Samples were analyzed for (**A**) Angiopoietin-2 (Ang-2), (**B**) Intercellular Adhesion Molecule 1 (ICAM-1) and (**C**) Vascular Cell Adhesion Molecule 1 (VCAM-1) protein content. Abbreviations: K – control CMV, T – TMV, P – septic SMV, C – patient CMV.
